# Determinants of Adolescent Sexual Behavior in Sub-Saharan Africa: A Systematic Review of In-School and Out-of-School Adolescents

**DOI:** 10.3389/phrs.2025.1608357

**Published:** 2025-09-23

**Authors:** Ruth-Janet Koumba Maguena, Sara Alves Jorge, Edgard Brice Ngoungou, Stephan Van den Broucke

**Affiliations:** ^1^ Institut de Recherche en Sciences Psychologiques, Université Catholique de Louvain, Louvain-la-Neuve, Belgium; ^2^ Institut de Recherche Santé et Société, Université Catholique de Louvain, Brussels, Belgium; ^3^ Unité de Recherche en Épidémiologie des Maladies Chroniques et Santé Environnement, Université des Sciences de la Santé, Libreville, Gabon; ^4^ Département de Médecine Communautaire ou Sociales, Université des Sciences de la Santé, Libreville, Gabon

**Keywords:** adolescent behavior, sexual behavior, sexual violence, determinants of health, sub-Saharan Africa

## Abstract

**Objectives:**

This review aims to identify the main determinants of various sexual behaviors among in-school and out-of-school adolescents in sub-Saharan Africa, using Bronfenbrenner’s ecological system theory as a framework.

**Methods:**

A systematic review with narrative synthesis was performed of empirical studies about the determinants of sexual behavior among in-school and out-of-school adolescents aged 10 to 19 in sub-Saharan Africa, using quantitative data from Embase, PubMed, PsycInfo, SCOPUS, AJOL, and Google Scholar.

**Results:**

A total of 132 studies were retrieved, identifying determinants of sexual behaviors such as sexual intercourse, sexual initiation at early age, multipartnership, condom use, contraceptive use, sexual abstinence, or sexual violence. Only four of these 132 studies involved out-of-school adolescents. Determinants of adolescent sexual behavior include demographic characteristics, socio-cognitive factors, problem behaviors, experiences, family relations, peers’ influence, relationships, school dynamics, education system, health system, information system, sociodemographic characteristics, socio-economic characteristics, religion, and societal values and norms.

**Conclusion:**

To design interventions that address adolescents’ sexual and reproductive health needs, public health policymakers must consider the multifaceted and interconnected determinants of sexual behaviors among in-school and out-of-school adolescents.

## Introduction

Sub-Saharan Africa (SSA) has the youngest population in the world, with 32% of the region’s population aged 10–24 years [1]. Investing in their health, education, employment, and civic participation could unlock significant development opportunities for these countries. However, these youth face many challenges particularly in education and sexual health. Of all regions of the world, SSA has the highest rates of education exclusion, with nearly 60% of young people aged 15 to 17 out-of-school [2]. Since adolescents are at high risk of sexually transmitted infections (STIs) and unintended pregnancies, their sexual health is a critical public health issue [3–5]. The World Health Organization (WHO) defines sexual health as “a state of physical, emotional, mental and social wellbeing in relation to sexuality; it is not merely the absence of disease, dysfunction or infirmity” [6]. Sexual health requires a positive and respectful approach to sexuality and sexual relationships, as well as the possibility of having pleasurable and safe sexual experiences, free of coercion, discrimination and violence. For sexual health to be attained and maintained, the sexual rights of every person must be respected, protected and fulfilled. The above definition of sexual health is a working definition, allowing for variations between countries to reflect their health priorities and social norms. It encompasses not only the biomedical aspects of sexual health but also the social and interpersonal dimensions essential to wellbeing.

Sexual health is intrinsically related to sexual behavior, which encompasses how individuals experience and express their sexuality. Sexuality is a natural and important part of being human, influencing nearly every aspect of one’s being, from attitudes and values to feelings and experiences [7]. Sexual behavior is a complex concept for which the literature does not provide a consensual definition. Consequently, it can be operationalized in several ways. Depending on the definition used, it comprises various sexual activities that involve sexual arousal, desire, as well as procreation, sexual risk behavior, sexual orientation, sexual violence, and many other aspects [8]. While the practice of sex education typically approaches sexual behavior primarily in terms of avoiding risky sexual behavior (e.g., early sexual initiation, condom use, multipartnership), education about sexuality can also be approached from a positive perspective. Positive sexuality means that one accepts and respects one’s sexuality and that of other persons without judgment, shame, violence, or discrimination [7]. An increasing number of studies assert that the experience of positive sexuality not only enhances sexual health but also mental health [8].

Since developing a healthy sexuality and healthy sexual behavior is a core developmental task, comprehensive sexuality education is most often focused on adolescents. Adolescence is the human development stage between childhood and adulthood, when people experience intense physical, cognitive, and psychosocial growth. During this phase, adolescents develop behavior patterns that influence their future health, either positively by promoting and protecting health or negatively by putting themselves and others at risk. To understand the determinants of adolescent behavior, Bronfenbrenner’s ecological systems theory offers a comprehensive framework by conceptualizing development as shaped by continuous interactions between individuals and their environment [9]. This model structures the environment into nested systems:

- The ontosystem refers to individual-level attributes (e.g., biology, cognition, attitudes).
- The microsystem encompasses immediate settings (e.g., family, peers, school) with which the individual interacts directly.
- The mesosystem represents interrelations among these immediate environments.
- The exosystem includes external contexts that indirectly influence the individual (e.g., parental workplace, local policies).
- The macrosystem refers to broader sociocultural and political contexts (e.g., norms, economic structures).
- The chronosystem accounts for the dimension of time and life-course transitions.

This ecological approach enables a multilevel analysis of behavioral determinants, from proximal to structural factors.

A large range of studies have been performed to identify the factors that influence specific sexual behaviors among youth in SSA. However, nearly all of these have focused on only a few specific behaviors and/or on a limited number of their determinants, while very few studies have tried to identify the broad range of determinants using an encompassing theoretical model. In addition, most research has been limited to the sexual behavior of in-school adolescents, despite the high rates of educational exclusion among adolescents in SSA countries. The present systematic review aimed to address these limitations by identifying and integrating the main determinants of a variety of sexual behaviors among both in-school and out-of-school adolescents in SSA, using Bronfenbrenner’s ecological system as a theoretical framework. The ultimate purpose was to provide a basis that would enable public health policymakers in SSA to develop more comprehensive and effective sexual health education interventions for adolescents.

## Methods

A systematic review was performed of empirical studies that produced quantitative data on the determinants of adolescent sexual behavior in SSA. A systematic review protocol following the Preferred Reporting Items for Systematic Review and Meta-Analyses (PRISMA) guidelines [10] was published in PROSPERO on December 3, 2022 (reference: CRD42022384658).

### Search Strategy

To find studies presenting evidence on determinants of in-school and out-of-school adolescent sexual behavior in SSA, the following databases were consulted: Embase, PubMed, PsycInfo, SCOPUS, AJOL, and Google Scholar. Search equations were built using descriptors and keywords related to adolescent, sexual behavior, determinant, and sub-Saharan Africa, organized with Boolean terms. Results were imported into Rayyan, a web-based tool for systematic literature review. After eliminating duplicate references, two independent reviewers (R.K. and S.A.) screened articles based on predefined eligibility criteria in two rounds: first by titles and abstracts, then by full texts. Database searches were performed on April 24, 2023, and on Google Scholar only on April 28, 2023. Reference lists of included studies were scanned to identify additional studies and ensure literature saturation.

### Eligibility Criteria

Studies were included in the systematic review if they: (1) were conducted in sub-Saharan countries; (2) focused on in-school or out-of-school adolescents aged 10–19 years (as defined by the United Nations (UN)), whereby the adolescent subpopulation had to be the majority (over 50%); (3) used quantitative methodology; (4) investigated determinants of various sexual behaviors; (5) were published between January 2010 and April 2023 (2010 being 1 year after the publication of the first edition of UNESCO’s International Technical Guide on Adolescent Sexuality Education, a crucial moment in the recognition of adolescents' right to sexuality education); (6) were published in English or French.

Studies were excluded when they: (1) failed to meet one or more of the above inclusion criteria; (2) lacked detail to verify majority of participants were 10–19 years old; (3) did not specify if participants were in-school or out-of-school; (4) did not explicitly address determinants of sexual behavior; (5) used only qualitative data. Conference abstracts, commentaries, and letters that did not contain sufficiently detailed information about the study were also excluded, as were studies for which the full text could not be retrieved, even contacting the corresponding author.

### Quality Assessment

To assess the methodological quality and risk of bias of the studies retained for the review, two independent assessors (R.K. and S.A.) scored all articles using the Joanna Briggs Institute (JBI)’s critical appraisal tool for analytical cross-sectional studies [11]. The JBI checklist considers 8 quality criteria: (1) definition of inclusion criteria, (2) description of study subjects and the setting, (3) valid and reliable measurement of the exposure, (4) objective criteria for measuring the condition, (5) identification of confounding factors, (6) strategies for dealing with confounding, (7) valid and reliable measurement of outcomes, and (8) appropriate statistical analysis. To assess the quality of each study, an overall score was calculated expressed as a percentage, with a score of 70%–100% indicating high methodological quality, a score of 50%–69% moderate methodological quality, and a score below 50% poor methodological quality with a high risk of bias. The quality assessment was used for indicative purpose only, meaning that no studies were excluded based on this assessment.

### Data Extraction and Synthesis

Data extraction was performed by a single reviewer (R.K.) using a standardized and pretested extraction form developed collaboratively by the research team. This form clearly defined data fields to ensure consistency in recording key study characteristics and findings. In addition, any uncertainties were resolved through discussion with the co-authors.

Due to the heterogeneity of definitions and measurements of sexual behaviors and their determinants across the included studies, a meta-analysis was not feasible. Instead, we conducted a narrative synthesis using a qualitative content analysis approach [12]. To reduce potential subjectivity, key decisions such as grouping different terminologies referring to the same sexual behavior or category of determinant were reviewed and validated in consultation with a second author (SV). For each category of sexual behavior, we listed the identified determinants and retained those most frequently reported across studies. Bronfenbrenner’s ecological model was applied deductively: determinants were categorized according to predefined ecological levels based on their nature and the context in which they were described. When difficulties or overlaps in classification arose, decisions were discussed among co-authors to ensure clarity and coherence in the allocation. Given the strong interconnections between determinants classified under the exosystem and the macrosystem, we decided to merge these categories and report all such determinants under the macrosystem level.

## Results

**FIGURE 1:**
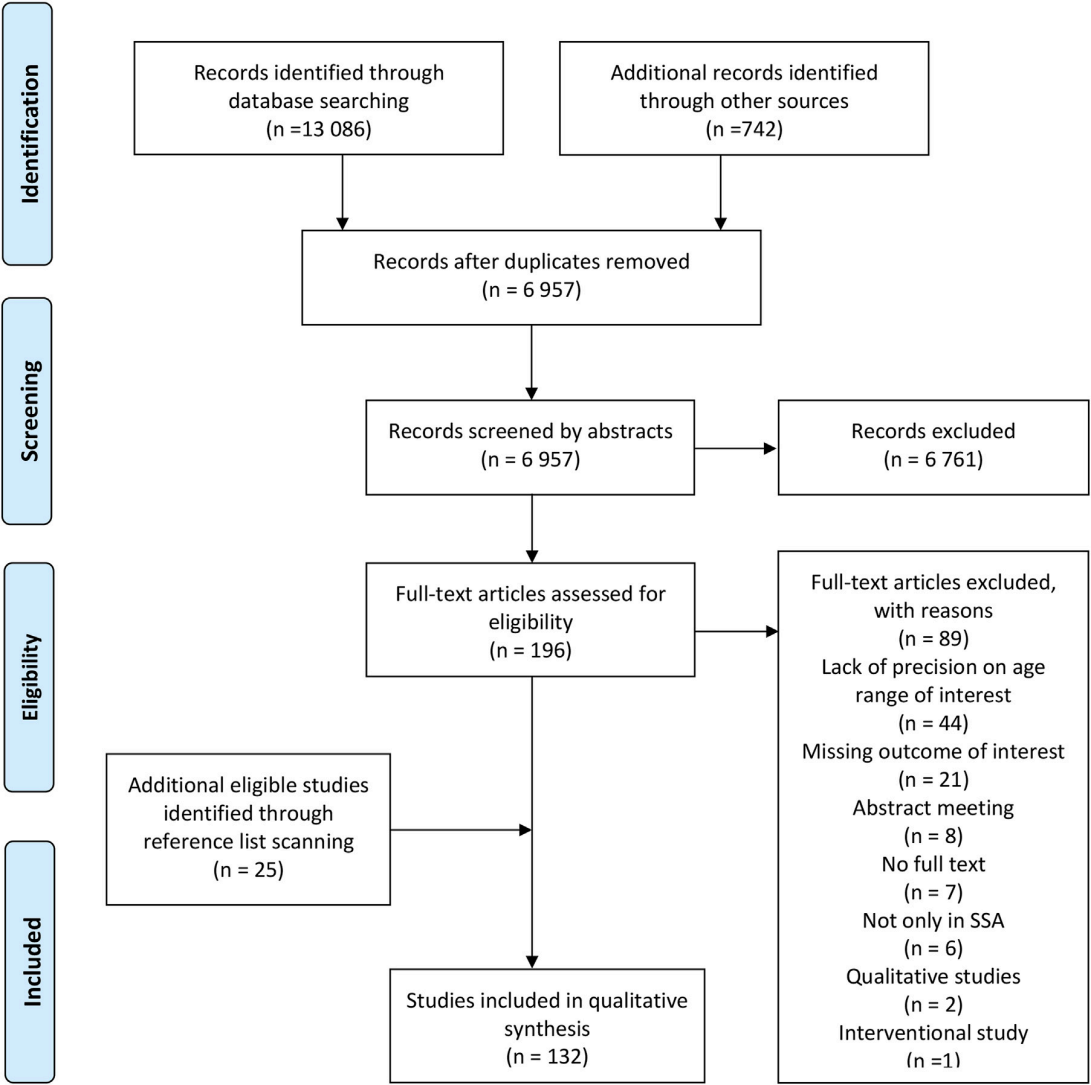
PRISMA flowchart of the study selection process (Determinants of adolescent sexual behavior in sub-Saharan Africa: a systematic review of in-school and out-of-school adolescents, sub-Saharan Africa countries, 2010–2023).

The search strategy produced 6957 records from different databases and reference lists, after removing duplicates. Screening based on titles and abstracts resulted in the exclusion of 6761 records, and another 89 records were excluded after screening the full texts, resulting in a total of 107 records for inclusion. The main reasons for exclusion of study records were a lack of precision regarding the proportion of the adolescent sub-population, not addressing the determinants of sexual behavior, unavailability of a full text, and not focusing on SSA. The reference lists of the 107 eligible studies were scanned to ensure literature saturation. Twenty-five additional studies were identified, giving a final total of 132 studies that met all the inclusion criteria and that were thus included in the content analysis and synthesis (Figure 1).

### Description of the Included Studies

The 132 studies included for this review covered 28 countries in Sub-Saharan Africa. Six studies contained data from several SSA countries, the other 126 studies had data from one country. Of these, a majority (over 70%) were concerned with one of three countries: Ethiopia (n = 60), Nigeria (n = 21), and South Africa (n = 17). Most studies involved adolescents from secondary or preparatory schools, while one study focused on university adolescent, two on out-of-school adolescents, and two on a mix of in- and out-of-school adolescents. All studies were cross-sectional, employing quantitative data collected via validated and non-validated questionnaires. Among them, 104 investigated determinants of risky sexual behaviors (RSB), and 28 investigated determinants of sexual violence (SV).

### Quality Assessment of Included Studies

The overall quality of the included studies was medium to strong, according to the assessment using the JBI critical appraisal tool. More precisely, 45 studies scored above 70% for overall quality, 75 studies scored between 50% and 70%, and 12 studies scored below 50%. The main limitations to quality were a lack of clear information on participant inclusion, a lack of identification of confounding factors, a lack of an explicit strategy for dealing with the effect of confounding factors, and a lack of validation of the questionnaires that were used.

### Determinants of Risky Sexual Behaviors

A first series of findings concerns the main determinants of sexual behaviors that can be considered as risky, due to their potential to increase the risk of STIs or unintended pregnancies among adolescents. Risky sexual behavior includes sexual intercourse, premarital sex, early sexual intercourse, having multiple sexual partners, not using contraceptives, and not using condoms. Because of the interconnectedness of these risk behaviors, most studies used measures that considered all these types of RSB together. Of the 104 included studies, 101 involved school-going adolescents, one involved out-of-school adolescents, and two involved both.

Determinants of RSB are factors that can either protect against RSB or increase the probability of RSB. Based on Bronfenbrenner’s ecological systems theory, they can be categorized as related to the adolescent’s ontosystem, microsystem, mesosystem, and macrosystem (Table 1).

**TABLE 1:** Determinants of risky sexual behaviors (Determinants of adolescent sexual behavior in sub-Saharan Africa: a systematic review of in-school and out-of-school adolescents, sub-Saharan Africa countries, 2010–2023).

	Factors protecting against risky sexual behavior	Factors increasing the probability of risky sexual behavior	Ambivalent findings
**ONTOSYSTEM**			
Individual demographic characteristics	- Being single [13–15] - Being religious [15–20] - Being Protestant (compared to Catholics or Muslims) [21] - High level of parental education [22–30]	- Being in a relationship [27, 28, 31–40] - Not being religious [22, 41–44] - Being Catholic (compared to Muslims) [21, 23, 45] - Rural residence [22, 36, 37, 46–49] - Living without families [23, 25, 41–43, 50–52] - Living alone [51, 53] - Living with a single parent [54]	- Male gender [22, 31, 32, 41, 50, 51, 54–66] - Female gender [13, 23–27, 33, 45, 46, 53, 67–69] - Age [16–18, 21, 22, 33–39, 42, 43, 45, 47, 51, 57, 58, 60, 61, 63–66, 70–78] - Ethnicity [18, 61, 79] - Grade level [14, 19, 22, 23, 31, 38, 53, 55, 78, 80–84] - Urban residence [31, 43, 50, 53, 85]
Socio-cognitive factors	- Good knowledge of STIs, HIV, and safe sexual practices [17, 28, 42, 56, 64, 82, 86, 87] - Self-efficacy in adopting safe sexual behaviors [13, 18, 53, 79] - Empowerment in safe sexual behaviors [88] - Positive attitude towards safe sexual behaviors and abstinence [13, 86, 88] - High perceived risk associated with risky sexual behavior [18, 70]	- Perceived peer pressure [16, 23, 26, 36, 38, 41, 43, 47, 48, 50, 55, 56, 58, 61, 62, 67, 82, 83, 89, 90] - Perceived gender norms [58, 70] - Perception of social norms associated with sexual behaviors of peers and family [17, 47, 61, 70, 91] - Low perception of social norms regarding risky sexual behavior and abstinence [64, 73, 87, 92] - Low perceived risk associated with risky sexual behavior [21, 79] - Low intention to adopt safe sexual behaviors [73] - Positive attitude towards risky sexual behaviors [93]	
Behaviors		- Substance use [55, 57], [50, 58–61], [31, 32], [22, 65, 66], [13, 23–26], [46], [45, 67, 68], [16, 19, 36, 71, 74, 81, 83, 84], [28–30, 48], [52, 86, 89, 90, 92], [40, 44, 94–98] - Pornography use [14, 16, 22, 27–29, 32, 36, 40, 41, 46, 48–51, 67, 68, 80, 82, 90, 98, 99] - Internet use [33, 89, 95] - Risky sexual behaviors [21, 48, 50, 63, 74, 93] - Sexting [99] - Partying [37, 68, 89, 100] - Violent behavior [20, 101] - Truancy [84]	
Experiences	- Experience in romantic relationships increasing [102, 103]	- Sexual harassment [76, 79] - Sexual violence [76] - Domestic violence [23] - Parental neglect [52] - Knowing someone who has died of HIV [79]	
**MICROSYSTEMS**			
Family	- Good family connectedness [19, 25, 44, 58, 84, 87, 91, 104] - Discussions on sexual and reproductive issues [26, 27, 49, 56, 80, 98] - Good parental monitoring [26, 38, 94, 104] - Authoritative parenting style [87] - Family support [44, 63, 65, 105] - Living parents [35, 106]	- Poor parental monitoring [48, 54, 55, 67, 75, 98] - Lack of discussion on sexual and reproductive health issues [14, 23, 24, 54, 82, 90] - Polygamous families [42, 54, 75] - Family size in the household [83]	
Peers	- Having close friends [57, 84]	- Sexually active friends [16, 47, 59, 74, 78, 94]	Discussions on sexual and reproductive health issues [18, 22, 51, 62, 70, 81]
Relationship	- Being in a stable romantic or sexual relationship [85]		
School	- Good school connectedness [19, 44, 66, 78] - Good academic performance [34] - School attendance [65] - Ambitious educational goals [107]	- Dropping out of school [108]	
**MESOSYSTEM**			
Socio-economic characteristics	- Receiving little or no pocket money [24, 50, 80]	- Low socio-economic status family [72] - Receiving money from family [27, 32, 42, 53, 62, 81] - Earning money through working [27, 76, 109] - Receiving money from a boyfriend [76]	- High socio-economic status family [51, 102]
Social interactions	- Social cohesion [69]	- Social support [52, 61, 104] - Social trust [69]	
**MACROSYSTEM**			
Education system	- Religious school [40, 67] - Access to sexual and reproductive health programs [79, 92, 110]	- Public school [99] - Mixed school [35]	
Health system	- Access to health services [105]		
Information system	- Awareness campaigns [18, 79]		- Access to information about sexual and reproductive health issues [14, 42]

At the ontosystem level, determinants include demographic characteristics, socio-cognitive factors, behaviors, and exposure to specific experiences. Findings for several sociodemographic characteristics are ambivalent. Being male [22, 31, 32, 41, 50, 51, 54–66] or female [13, 23–27, 33, 45, 46, 53, 67–69], age [16–18, 21, 22, 33–39, 42, 43, 45, 47, 51, 57, 58, 60, 61, 63–66, 70–78], ethnicity [39, 49, 50], grade level [14, 19, 22, 23, 31, 38, 53, 55, 78, 80–84], and urban residence [31, 43, 50, 53, 85] are identified both as factors increasing and reducing the probability of RSB. Being single [13–15], religious [15–20], and having parents with a high educational level [22–30] are protective factors against RSB. Conversely, rural residence [22, 36, 37, 46–49], living without families [23, 25, 41–43, 50–52], alone [51, 53], or with a single parent [54] increase the probability of RSB. Good knowledge of STIs, HIV, and safe sexual practices is mostly protective [17, 28, 42, 56, 64, 82, 86, 87], except in one study [73]. Self-efficacy in adopting safe sexual behaviors [13, 18, 53, 79], empowerment in safe sexual behaviors [88], a positive attitude toward safe sexual behaviors and abstinence [13, 86, 88], and a high perception of risks associated with RSB [18, 70] are protective factors. Perceived peer pressure generally increase the probability of RSB [16, 23, 26, 36, 38, 41, 43, 47, 48, 50, 55, 56, 58, 61, 62, 67, 82, 83, 89, 90], except in one study [18]. Perceived gender norms [58, 70], perception of social norms associated with the sexual behavior of peers and family [17, 47, 61, 70, 91], a low perception of social norms regarding RSB and abstinence [64, 73, 87, 92], a low perception of risks associated with RSB [21, 79], a low intention to adopt safe sexual behaviors [73], and a positive attitude towards RSB increase the probability of RSB [93]. Behaviors such as substance use [55, 57], [50, 58–61], [31, 32], [22, 65, 66], [13, 23–26], [46], [45, 67, 68], [16, 19, 36, 71, 74, 81, 83, 84], [28–30, 48], [52, 86, 89, 90, 92], [40, 44, 94–98], except in one study [34], pornography use [14, 16, 22, 27–29, 32, 36, 40, 41, 46, 48–51, 67, 68, 80, 82, 90, 98, 99], internet use [33, 89, 95], sexting [99], partying [37, 68, 89, 100], violent behavior [20, 101], and truancy [84] increase the probability of RSB. Experiences of sexual harassment [76, 79], sexual violence [76], domestic violence [23], parental neglect [52], and knowing someone who died of HIV [79] also increase the probability of RSB.

At the microsystems level, family, peers, relationships, and school factors influence RSB. Discussions on sexual and reproductive health (SRH) issues with family are mainly protective against RSB [26, 27, 49, 56, 80, 98], except in one study [47]. Discussions with peers can either increase or reduce the probability of RSB [18, 22, 51, 62, 70, 81]. Good family connectedness [19, 25, 44, 58, 84, 87, 91, 104], parental monitoring [26, 38, 94, 104], family support [44, 63, 65, 105], having living parents [35, 106], close friends [57, 84], being in a stable relationship [85], good school connectedness [19, 44, 66, 78], academic performance [34], and ambitious educational goals [107] are protective factors. Conversely, having sexually active friends [16, 47, 59, 74, 78, 94], polygamous families [42, 54, 75], large household size [83], and dropping out of school [108] increase the probability of RSB.

At the mesosystem level, socio-economic characteristics and social interactions influence RSB. Coming from a low socio-economic family increases the probability of RSB [72], while results for high socio-economic family are ambivalent [51, 102]. Receiving money from family [27, 32, 42, 53, 62, 81], a job [27, 76, 109], or a boyfriend [76] increases the probability of RSB, whereas not receiving money is protective.

Finally, at the macrosystem level, religious schools are protective against RSB [40, 67], while public [99] and mixed schools [35] increase the probability of RSB. The influence of access to information on SRH issues depends on the source [14, 42]. School-based SRH programs [79, 92, 110], health services [105], and awareness campaigns [18, 79] are protective factors against RSB.

### Determinants of Sexual Violence Victimization and Perpetration

The second series of findings concerns studies that focus on the determinants of sexual violence among adolescents in SSA, i.e., the risk and protective factors of either victimization or perpetration of SV. The WHO defines sexual violence as “any sexual act, attempt to obtain a sexual act, or other act directed against a person’s sexuality using coercion, by any person regardless of their relationship to the victim, in any setting” [111]. Due to the interrelationship between sexual violence and psychological or physical violence, some studies consider all these types of violence together.

Of the 28 included studies that dealt with sexual violence (Table 2), only one involved out-of-school adolescent girls. Eleven studies focused exclusively on girls who were victims of SV, one on boys who were victims, and three on boys who were perpetrators.

**TABLE 2:** Determinants of sexual violence victimization and perpetration (Determinants of adolescent sexual behavior in sub-Saharan Africa: a systematic review of in-school and out-of-school adolescents, sub-Saharan Africa countries, 2010–2023).

	Factors protecting against sexual violence victimization	Factors increasing the probability of sexual violence victimization	Factors protecting against sexual violence perpetration	Factors increasing the probability of sexual violence perpetration
**ONTOSYSTEM**				
Individual demographic characteristics	- High grade level among girls [112] - Being Muslims (compared to Christians) among girls [113] - Living with both parents among girls [114]	- Being older [115–117] - Being in a relationship [117–120] - Lack of religions commitment [121] - Living alone or with friends [122–124] - Living with a single parent [112, 122] - Living with family members [113, 124] - Living with a boyfriend [39] - Rural residence [117] - Living in middle-class or lower-class districts [112]	- Female gender [125]	- Male gender [115, 116, 125] - Being older [115, 116, 125] - Being in a relationship [126] - Low grade level [115]
Socio-cognitive factors	- Generalized self-efficacy among girls [127, 128]	- Poor knowledge of child rights among girls [129] - Perceived gender norms [118, 127] - Perceived peer pressure to have sex [130] - Perceived risk to contract HIV [115] - Positive attitude towards male sexual entitlement [131] - Tolerance towards sexual violence [130]		- Poor knowledge of child rights among girls [129]
Behaviors		- Substance use among girls [107, 122, 128, 130, 132] - Pornography use among girls [130] - Having perpetrated sexual violence [115, 125, 133] - Risky sexual behaviors [115, 120, 126, 130, 132–134]		- Substance use among boys [126, 135] - Pornography use among boys [126] - Having perpetrated sexual violence [115, 125, 133] - Risky sexual behaviors [115, 120, 126, 130, 132–134]
Experiences		- Experienced psychological, physical, and/or sexual violence [115, 125, 127–129, 132, 136, 137] - Domestic violence [119, 120, 128] - Being bullied [118, 132] - Having had a breakup [93]		- Experienced psychological, physical, and/or sexual violence [115, 125, 127–129, 132, 136, 137]
**MICROSYSTEMS**				
Family		- Polygamous families [118] - Separated or divorced parents [114] - Parental conflict [118] - Fearful parental attachment among girls [130] - Lack of discussion on sexual and reproductive health issues among girls [122, 123]		- Discussion on sexual and reproductive health issues among boys [126]
Peers		- Sexual violence victimization and/or perpetration by peers [129]		- Sexual violence victimization and/or perpetration by peers [129]
Relationship		- Having an older partner [130, 134] - Communication with male partner among girls [132]		
School	- Never attending school among girls [138]	- Low school connectedness [125] - Low feelings of safety at school [125] - Poor school engagement among girls [132] - Schoolwork problems [118] - Having repeated a school year [125]		- Low school connectedness [125] - Low feelings of safety at school [125] - Poor school engagement among boys [132]
**MESOSYSTEM**				
Socio-economic characteristics	- Father working full-time, part-time or retired among girls [112, 114]	- Low socio-economic status family [116, 123] - Unemployed head of household [121], - Receiving money from external sources [117] - Food deprivation [129, 139]		- Low socio-economic status family [116] - Food deprivation [129, 139]
Social interactions				- Low social support [115]
**MACROSYSTEM**				
Education system	- Private school among girls [113]	- Public school among girls [120]		- Boarding school [115]
Health system	- Financial accessibility to health services [128]			
Social values and norms		Unequal and sexist gender norms [129]		Unequal and sexist gender norms [129]

At the ontosystem level, determinants include demographic characteristics, socio-cognitive factors, behaviors, and exposure to specific experiences. The influence of gender on SV victimization is ambivalent [115–118, 140], but being male increases the probability of SV perpetration [115, 116, 125], while being female is protective [125]. Being older [115–117, 125] and in a romantic relationship [117–120, 126] increase the probability of both SV victimization and perpetration. Living with both parents is protective against SV victimization [114], while other living arrangements [39, 112, 113, 122–124] increase SV victimization. Poor knowledge of child rights among girls increases the probability of both SV victimization and perpetration [129]. Perceived gender norms [118, 127], perceived peer pressure to have sex [130], perceived risk of contracting HIV [115], positive attitudes toward male sexual entitlement [131], and tolerance towards SV [130] increase the probability of SV victimization, while generalized self-efficacy is protective. Substance use [107, 122, 126, 128, 130, 132, 135], pornography use [126, 130], previous SV perpetration [115, 125, 133], and experiencing any type of SV [115, 125, 127–129, 132, 136, 137] increase both SV victimization and perpetration. Witnessing domestic violence [119, 120, 128], being bullied [118, 132] and having experienced a breakup [93] increase only SV victimization.

At the microsystems level, family, peers, relationship, and school factors influence SV victimization and perpetration. Polygamous families [118], separated parents [114], parental conflict [118], and fearful parental attachment among girls [130] increase SV victimization. Discussions about SRH issues with family increase SV perpetration among boys [126], while lack of discussion increases SV victimization among girls [122, 123]. Peers’ SV experience [129], low school connectedness [125], low feelings of safety at school [125], and poor school engagement [132] increase both SV victimization and perpetration. Having an older partner [130, 134], schoolwork problems [118], and having repeated a school year [125] increase SV victimization, while never attending school is protective for girls [138].

At the mesosystem level, socio-economic characteristics and social interactions influence SV. Low socio-economic status family [116, 123] and food deprivation [129, 139] increase both SV victimization and perpetration. An unemployed head of household [121] and receiving money from external sources [117] increase SV victimization, while a father working, or retired is protective for girls [112, 114].

Finally, at the macrosystem level, educational system, health system, and societal values and norms influence SV. Attending public school increases SV victimization among girls [120], while private school is protective [113]. Boarding school increases SV perpetration [115]. Financial access to health services is protective against SV victimization [128]. Unequal and sexist gender norms increase both SV victimization and perpetration [129].

## Discussion

This study aimed to identify and synthesize the main determinants of in-school and out-of-school adolescent sexual behavior in SSA, focusing on the risk and protective factors for risky sexual behaviors and for victimization or perpetration of sexual violence. The results showed that the sexual behavior of adolescents in SSA is influenced by a multitude of interconnected determinants. Based on Bronfenbrenner’s ecological model, we organized these determinants into four levels and identified key implications for interventions at each.

At the ontosystem level, apart from several demographic characteristics, the main determinants identified include socio-cognitive factors such as knowledge, attitudes and perceived norms. While good knowledge about SRH protects adolescents from RSB and SV, beliefs and emotional factors often override rational decision-making. Interventions should therefore not only ensure access to reliable information from key sources (peers, family, school, media, and professionals [141]), but also address attitudes and perceived norms, particularly those promoting RSB and SV. According to health behavior theories such as Ajzen’s Theory of Planned Behavior (TPB) [142–145], sexual behavior is shaped by behavioral intentions influenced by attitudes, perceived norms, and perceived behavioral control. Our review showed that positive attitudes toward protective sexual practices and abstinence are associated with refraining from RSB, while favorable attitudes towards RSB and SV promote these harmful behaviors. Moreover, limited perceived risk related to sexual behaviors and perceived peer pressure to have sexual intercourse also contribute to RSB and SV, whereas self-efficacy empowers adolescents to adopt protective sexual behaviors. These findings are consistent with the Protection Motivation Theory (PMT) [146], which also emphasizes the role of cognitive appraisal in behavior change. Therefore, interventions should incorporate socio-cognitive approaches, such as the TPB or PMT, to strengthen behavioral intentions toward safer sexual practices. Our review also found that RSB often co-occur with other problem behaviors [147], including substance use, internet and pornography use, sexting, partying, violent behavior, and truancy. Substance use may contribute to RSB through mechanisms like disinhibition [148], while exposure to pornography, particularly when it portrays non-consensual or aggressive acts, can distort adolescents’ perceptions of appropriate sexual behavior [149]. Therefore, integrated interventions should address these co-occurring risk behaviors, which heighten adolescents’ vulnerability to RSB and SV. Experiences of harassment, sexual and domestic violence, or parental neglect increase adolescents’ risk of RSB and SV by contributing to the normalization and potential reproduction of these patterns later in life [150]. These findings highlight the need for individual-level interventions that identify and address adolescents’ traumatic experiences as part of comprehensive RSB and SV prevention strategies in SSA.

At the microsystems level, the review highlights the crucial role of interpersonal relationships with family, peers, partners, and within schools in shaping adolescent sexual behavior. A supportive family environment with present parents, adequate supervision, strong relationships, and open communication on SRH creates a protective environment against RSB and SV. Strengthening parent-child communication and encouraging parental involvement should therefore be central to SRH interventions [151]. Peers also influence adolescent behavior, acting as role models who normalize either risky or protective practices [152]. This influence can be leveraged through peer education strategies that promote safe sexual behaviors [153]. Romantic relationships are another important dimension. Adolescents in relationships are more likely to have early sexual relations and use contraceptives, but large age gaps increase the risk of SV [154]. Relationship stability, exclusivity, communication, mutual respect, and equitable power dynamics play a key role in shaping healthy sexual behavior. Promoting communication, respect, consent, and age-appropriate partnerships helps prevent risky situations and coercion. More broadly, educating adolescents about healthy interpersonal relationships is essential to reducing vulnerability. Finally, school connectedness plays a protective role. Adolescents who are engaged in school and have ambitious educational goals tend to show fewer RSB and SV. Interventions that support school attendance and strengthen students’ connection to school can thus reduce exposure to risky behaviors [155, 156].

At the mesosystem level, socioeconomic factors significantly influence adolescent sexual behavior, particularly when financial insecurity leads youth to seek material support through sexual exchanges. Adolescents from higher socio-economic backgrounds are less likely to engage in RSB and SV, likely due to better access to sexual education and SRH services, and reduced reliance on transactional relationships [157, 158]. In contrast, early financial independence may increase exposure to high-risk environments, such as unsupervised parties involving alcohol. Policies that reduce poverty and promote vocational training, youth employment, and economic empowerment can help reduce economic vulnerability and, in turn, risky behaviors. Interventions should also consider the risks associated with premature financial autonomy, which may facilitate engagement in unsafe practices.

At the macrosystem level, broader structural and cultural factors, including the education and health systems, media, and community norms, significantly shape adolescent sexual behavior. Access to health and social services remains uneven, particularly between urban and rural areas [159]. Likewise, the type of school and the availability of comprehensive sexuality education influence RSB and SV, with well-organized and equipped schools offering a more protective environment [156]. Providing adequate resources and expanding access to comprehensive sexual education in all schools, especially in public and rural settings, should therefore be a policy priority. Media, as a primary source of SRH information for adolescents [141], should be actively engage in promoting safe sexual practices and raising awareness around SV. Ethnic, religious, and social values and norms that characterize a community are also important determinants of adolescent sexual behavior. Religious beliefs promoting abstinence may act as protective factors, particularly when religiosity is strong [160], suggesting that partnerships with religious communities could help promote safer sexual practices. Conversely, traditional gender norms that emphasize male dominance and stereotypical masculinity are associated with increased RSB and SV. Community-based interventions that aim to challenge and deconstruct these norms could therefore contribute to improving adolescent sexual health [161].

While this review aimed to include both in-school and out-of-school adolescents, the overwhelming predominance of studies on in-school youth reflects a major gap in the literature. Only four of the 132 included studies focused on out-of-school adolescents. Yet, the limited available data suggest that this group may face heightened vulnerability due to intersecting socioeconomic, educational, and familial disadvantages, which can increase exposure to RSB and SV. This imbalance limits the generalizability of our findings and highlights a critical evidence gap. More research is needed to identify and understand the specific determinants affecting out-of-school adolescents and to design targeted, inclusive interventions for this population.

### Limitations

This study is not without limitations. Most included studies relied on self-reported data, introducing potential social desirability and recall biases that could lead to under- or over-reporting of certain sexual behaviors, especially given the sensitivity of the topic. All studies used cross-sectional designs, limiting the ability to establish causal relationships. A major limitation of the literature is the strong imbalance between studies conducted among in-school and out-of-school adolescents affecting generalizability. Similarly, studies on SV victimization predominantly focused on girls, contributing to gender gaps in the evidence base. Although this review includes studies from various SSA countries, a large proportion of the data comes from a limited number of nations, notably Ethiopia, Nigeria and South Africa. This geographical concentration may limit the regional representativeness of the findings, as sociocultural, educational, and policy contexts vary widely across the continent. Finally, heterogeneity in definitions and measures of sexual behaviors and their determinants precluded the possibility of conducting a meta-analysis.

## Conclusion

Despite the limitations, this study provides a comprehensive overview of the determinants of various sexual behaviors among in-school and out-of-school adolescents in SSA, using Bronfenbrenner’s ecological model to identify and structure these determinants. It highlights the crucial role of socio-cognitive factors such as attitudes, perceived social norms, perceived behavioral control, and intention in shaping the adolescents’ sexual behaviors, while also considering risk perception, co-occurring behaviors like substance or pornography use, and experiences of harassment, sexual and domestic violence as key determinants. At the immediate environment level, family dynamics, peer and partner influences, and the school environment are primary determinants, while in the broader environment, community values and norms, socio-economic status, access to SRH services, and education (including comprehensive sexual education) are key influencing factors. Translating these findings into concrete strategies could strengthen both school-based and community-based interventions, contributing to improved adolescents’ SRH outcomes.

High rates of school exclusion in SSA call for inclusive and comprehensive sex education strategies that reach all adolescents, regardless of their schooling status. However, research and policy remain largely focused on school-going adolescents. It is therefore essential to develop targeted interventions addressing the unique challenges faced by out-of-school adolescents.

In conclusion, a holistic approach that integrates individual, interpersonal, socio-cultural, socio-economic, and contextual determinants is essential for designing effective sexual health interventions for adolescents in SSA. By addressing these determinants, public health policymakers will be better equipped to enhance the sexual health and overall wellbeing of adolescents in SSA.

## Abbreviations

HIV, Human Immunodeficiency Virus; PMT, Protection Motivation Theory; RSB, Risky Sexual Behavior; SRH, Sexual and Reproductive Health; SSA, Sub-Saharan Africa; STIs, Sexually Transmitted Infections; SV, Sexual Violence; TPB, Theory of Planned Behavior; WHO, World Health Organization.
